# Scientific Data Spaces - Experiences from the EGI-ACE project

**DOI:** 10.12688/openreseurope.17418.1

**Published:** 2024-07-09

**Authors:** Gergely Sipos, Giuseppe La Rocca, Fabrizio Antonio, Donatello Elia, Paola Nassisi, Sandro Fiore, Raul Bardaji, Ivan Rodero

**Affiliations:** 1 The EGI Foundation, Amsterdam, North Holland, The Netherlands; 2 CMCC Foundation - Euro-Mediterranean Center on Climate Change, Lecce, Italy; 3 University of Trento, Trento, Italy; 4 European Multidisciplinary Observatory of the Seabed and Water Column, Rome, Lazio, Italy

**Keywords:** Data Spaces, European Strategy for Data, EGI Federated Cloud, EMSO ERIC, ENES, GBIF, LOFAR, SeaDataNet

## Abstract

This paper presents the approach adopted by the EGI-ACE project for the setup and delivery of Data Spaces for various scientific domains. The work was implemented by members of the EGI e-infrastructure and of several European Research Infrastructures in the context of the European Open Science Cloud programme. Our results are several Data Space services that enable the reuse and exploitation of open, scientific big data for compute intensive use cases. The paper illustrates the EGI-ACE approach through two examples: (1) EMSO ERIC Data Portal for seafloor and water column research and (2) ENES Data Space for climate research.

## Plain language summary

The EGI-ACE project created a number of Data Spaces for various scientific domains, allowing for easy access and reuse of open, scientific big data for compute intensive use cases. The aim of the European Commission's strategy for data is to create a common European Data Space, which will bring together relevant data infrastructures and governance frameworks to facilitate data sharing while ensuring control for individuals and companies generating the data. The Data Spaces created within EGI-ACE contribute towards achieving this goal, publishing curated data from international scientific communities 'in the cloud' and offering them for scalable exploitation and reuse through domain-specific tools and environments that offer data visualization, analysis, and mapping. The paper provides an overview of the technical and policy implementation approaches of the EGI-ACE Data Spaces and their potential future role in the single market for data in Europe. The paper presents the generic approach that EGI-ACE followed for the implementation of scientific data spaces, and illustrates this with two examples, one from climate sciences (ENES), one from marine sciences (EMSO).

## 1. Introduction

The European Commission (EC) adopted a European strategy for data in 2020 with the aim of creating a single market for data that will ensure Europe’s global competitiveness and data sovereignty. This single market is referred to as “a common European Data Space” that brings together relevant data infrastructures and governance frameworks in order to facilitate data pooling and sharing. Common European Data Spaces will increase the availability of data for economic, societal, and research purposes, while ensuring that the companies and individuals who produce the data maintain control over it. Since 2020 the Commission has launched a series of projects to develop Data Spaces in strategic economic sectors and domains of public interest: Green Deal, Mobility, Health, Industry and Manufacturing, Financial Services, Energy, Agriculture, Public Administration. These sectoral projects will all lead towards individual Data Spaces that together, in the long term will formulate the common European Data Space.

EGI-ACE ran as a 30-month project between 2020–2023 in the European Commission co-funded European Open Science Cloud (EOSC) programme within Horizon Europe. EGI-ACE was coordinated by the EGI Foundation, with a consortium that included over 30 national compute centres from the EGI e-infrastructure federation, and 17 scientific institutes, Research Infrastructures and research communities - many present on the ESFRI roadmap.

One specific stream of work in EGI-ACE was the setup of scientific Data Spaces. These Data Spaces publish existing, curated data from international scientific communities ‘in the cloud’ (more precisely in a federation of clouds), and offer them for scalable exploitation and reuse through domain-specific tools and environments that offer data visualisation, analysis, mapping, etc. The Data Spaces are made accessible for users as online services through the EOSC Portal. (Remark: Since April 2024 the EOSC Portal is available as a snapshot archive. The European Commission is working on a new EOSC Portal and it is expected for live release during 2024).

This paper introduces the scientific Data Spaces that were enabled by EGI-ACE and provides details on their technical and policy implementation approaches. Section 2 provides an overview of related work, i.e. the current status of data spaces in Europe. Section 3 introduces the EGI-ACE Data Spaces and describes the common building blocks that enable them. Section 4 describes two of the EGI-ACE Data Spaces in detail. Section 5 closes with an outlook for the future, particularly how the EGI-ACE Data Spaces could become part of the single market for data in Europe.

## 2. Related work

The European strategy for data document defined key features of a common European Data Space, namely:

- A secure and privacy-preserving infrastructure to pool, access, share, process and use data.
- A clear and practical structure for access to and use of data in a fair, transparent, proportionate and/non-discriminatory manner and clear and trustworthy data governance mechanisms.
- European rules and values, in particular personal data protection, consumer protection legislation and competition law, are fully respected.
- Data holders will have the possibility, in the Data Space, to grant access to or to share certain personal or non-personal data under their control.
- Data that is made available can be reused against compensation, including remuneration, or for free.
- Participation of an open number of organisations/individuals.

Since 2020 the Commission has launched a series of projects to develop Data Spaces in strategic economic sectors and domains of public interest: Green Deal, Mobility, Health, Industry and Manufacturing, Financial Services, Energy, Agriculture, Public Administration. These projects are either in the landscaping phase, i.e. identification of the most relevant sectoral data providers, in the conceptualisation phase, i.e. defining suitable governance, business models and technologies for a sectoral data space, or in early implementation pilots of sectoral data spaces. Our EGI-ACE data spaces started earlier than these projects, and with timeline for setup of 30 months, shorter than the dedicated Data Spaces projects. However, our approach is also narrower in scope, to pre-identified data providers and with the use of pre-identified technologies from the EGI and scientific community software/service stacks (See Section 3 and Section 4 for details). 

Recently, the “European Data Spaces - Scientific Insights into Data Sharing and Utilisation at Scale” report ^1^ was released by the EC JRC, analysing the main EU policy documents to identify a set of key principles and high-level requirements for the Common European Data Spaces. The analysis highlights that, on one side, from a technical perspective, a single architecture or stack of technologies and standards cannot be universally applied. However, the document also recognizes that “a minimum stack of protocols and specifications […] is highly desirable” and the “forthcoming European Data Innovation Board, defined by the Data Governance Act and supported by the Data Spaces Support Centre (DSSC), should play a central role in the choice of such technologies and standards”.

An important step towards the minimal stack of common functionalities was achieved by the DSSC in September 2023, when the ‘ Data Spaces Blueprint v0.5’ ^2^ was released. In this document a data space is specified as a distributed system defined by a governance framework, that enables trustworthy data transactions between participants while supporting trust and data sovereignty. A data space is implemented by one or more infrastructures and supports one or more use cases.

The blueprint provides specification for data space building blocks, i.e. basic unit or component that can be implemented and combined with other building blocks to achieve the functionality of a data space. Two types of building blocks compose a data space: ‘business and organisational’ and ‘technical’. Our data space implementation also works with these two layers:

1. The building blocks of the business and governance layer are implemented by the governance of the EGI federation, and by the governance of the EGI-ACE project. While the EGI governance is responsible for the long-term strategy and membership, the project governance is responsible for the implementation aspect and for the day to day management of the Data Spaces including support for its users.
2. The technical building blocks are implemented by science domain specific, EGI-specific and EOSC-specific solutions using common approaches wherever possible among multiple Data Spaces. The common implementations are detailed in Section 3, the data space specific ones are detailed in Section 4.

**Figure 1.**
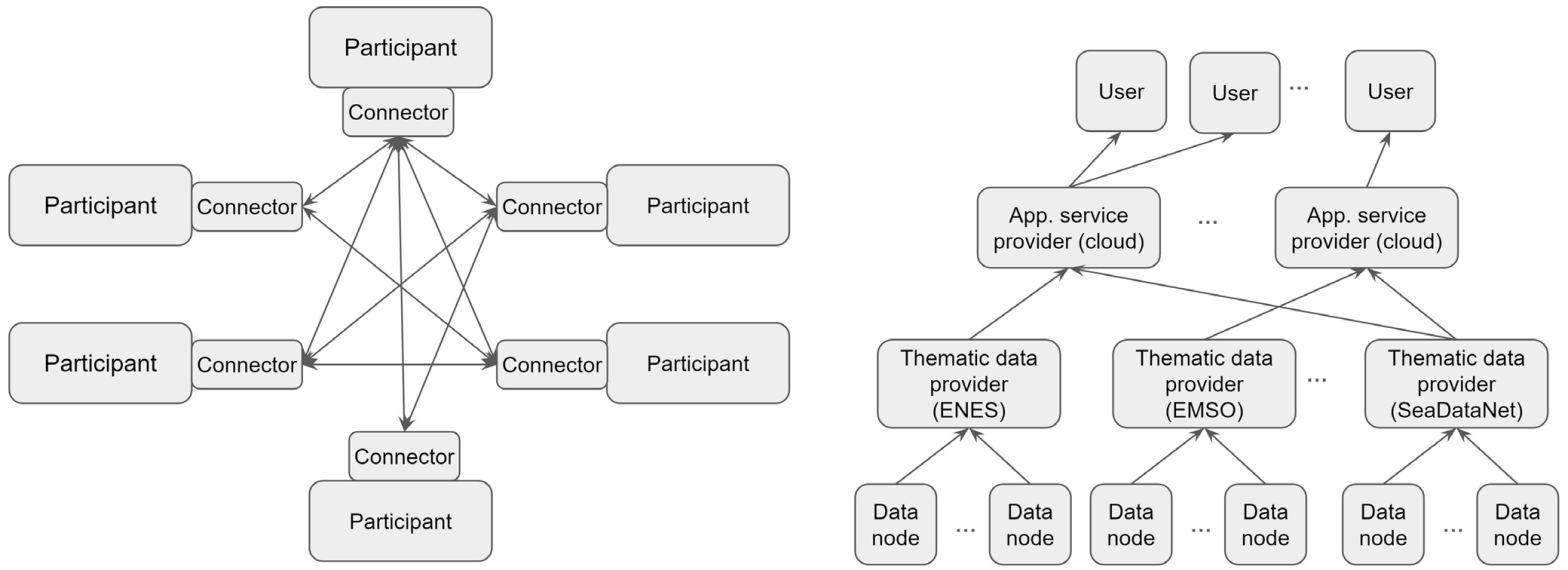
Architecture concepts for data spaces: DSSC (left), and EGI-ACE (right). Arrows indicate the direction of data flows.

While the Organisational and Technical layers are present in both our and the DSSC approaches, there are a number of differences between the DSSC Data Spaces concept and our implementation:

(1) DSSC describes Data Spaces as ecosystems that reach equilibrium without central planning. In our approach the EGI Foundation serves as a central coordinating entity that forms partnerships with data providers.
(2) DSSC expects that to achieve financial sustainability, a data space needs to attract use cases that generate income through data space usage fees. In our approach the cost of operation is covered by the providers from EC, national and institutional funds. The consumers benefit from free at point of use data and services in the data space.
(3) DSSC expects Data Spaces to grow in the number and types of use cases they can support. Our implementation is restricted to one specific type of use case, serving big scientific data through compute intensive applications.
(4) DSSC specifies Data Spaces as a set of ‘Participants’ each acting as both providers and consumers of data, with their transactions enabled by connectors. (See left side of Figure 1.) Our approach implements data provisioning towards one direction: from individual data provider nodes aggregated by thematic data providers to application providers (that run in cloud compute centres) and then to data consumers (scientists). (See right side of Figure 1).

## 3. EGI-ACE approach to scientific Data Spaces

EGI-ACE started just after the EC published the European strategy for data, with the overarching objective to enable researchers from any scientific discipline to perform data- and compute-intensive digital research through free-at-point-of-use, online services that span across the infrastructure, platform and application layers. The project consortium included 16 Cloud hosting nodes from the EGI Federation, 3 non-European e-Infrastructures (Open Science Grid from the US, CAS CNIC from China, IDIA from South Africa), 6 international communities of computational practice and/or data provisioning (LSGC, WeNMR, IS-ENES, SeaDataNet, Galaxy.eu, Disaster Mitigation Competence Centre), 10 Research Infrastructures (LOFAR, MeerKAT, EMSO, GBIF, ITER, EISCAT, VIRGO, e-RIHS, PHIRI, and OPERAS).

The project implemented and supported diverse types of computational use cases, all facilitating Open Science. One group of EGI-ACE use cases was called ‘Data Spaces’. Data spaces in EGI-ACE were established as a partnership between (1) international data curator & data publisher communities, (2) cloud compute provider centres and (3) technology provider institutes. The project enabled 5 of such Data Spaces:

1. EMSO ERIC Data Portal for seafloor and water column research
2. ENES Data Space for climate research
3. GBIF Cloud Data Space for biodiversity research
4. LOFAR Science Processing data space for radio astronomy
5. SeaDataNet WebOcean Data Analysis for marine research

The participating data curator and publisher communities are international organisations coordinating a network of scientific institutes to collect, curate, annotate and make scientific data accessible for broad use/reuse. Their mission is collecting and improving the quality of data assets, then publishing the data for scientific reuse. Their scope before the start of the EGI-ACE project was limited to data dissemination, i.e. tagging the data with coherent metadata and making the data available for download through Web/API portals.

The EGI-ACE Cloud hosting nodes deliver compute and storage capacity for data processing, data storage, application hosting. They can scale up data analytics environments, scientific gateways and other added value services operated by research communities and SMEs and industry.

Technology providers develop software and/or operate online services that enable the management of scientific data in the cloud and enable scalable computing applications with proper user access control (authentication/authorization).

EGI-ACE brought together the most appropriate combination of these three groups of stakeholders for each Data Space. Each group established a computational platform that enabled the scalable exploitation of scientific data online, in the clouds that participate in that given group. The data curator & publisher institutes remained the owners and quality controllers of the data, but they staged replicas of the data into the cloud centres. The cloud centres provided a stable and scalable platform to run applications that understand and can process the data of the given data space. The technology providers supported the setup with software services and technologies that for example reliably replicate subsets of the scientific data into OpenStack cloud storages, expose the data towards applications/portals that are running within the OpenStack sites, authenticate and authorise incoming users. 

EGI-ACE secured these partnerships with Service Level Agreements that specified the software, hardware and human capacities that the partners brought into the Data Space and offered a management framework for monitoring of the delivery, the Data Space uptake by users and its impact on science. The EC funding contributed not only to the time needed to reach the setup, but also funded the effort needed to serve international users once the Data Spaces were operational. The funding came in the form of ‘virtual access units’ that defined the cost of a ‘unit of access’ for a new user within the Data Space, and reimbursed this cost to the providers who enabled this access (to compute providers as well as to user supporters and trainers). The virtual access funding complemented existing national funds and increased service provider capacities so they could expand delivery of their services beyond their usual target groups (which are typically national users, or users in a given discipline). By combining national funds and international virtual access funds EGI-ACE was able to double the overall delivery capacity. (i.e. for every virtual access Euro provided by the European Commission EGI-ACE managed to obtain another Euro worth of service delivery capacity from the national sources of the service providers).

### 3.1 Compute technologies

The fundamental enabler of the EGI-ACE Data Spaces was the EGI Federated Cloud Compute service. This service provides a multi-cloud Infrastructure-as-a-Service (IaaS) federation, integrating OpenStack research clouds into a scalable computing platform suitable for data and compute-driven applications and services. It allows users to deploy and scale virtual machines on demand, offering computational resources in a secure and isolated environment managed through APIs, eliminating the need to handle physical servers. The clouds of the federation are harmonised from the user and managerial/operational perspectives, and form a federation relying on:

- A configuration database implemented by the EGI GOCDB system where each cloud can register its availability in the federation together with main characteristics, including the scientific communities they support.
- Single Sign-On implemented by the EGI Check-in service. Users can log into every provider with their institutional credentials (EduGAIN) and industry standards like OpenID Connect.
- Global VM image catalogue implemented by the EGI AppDB service. The catalogue provides pre-configured Virtual Machine images that are automatically replicated to every federated cloud site based on SLAs signed with each supported community.
- Global accounting that aggregates usage accounting (CPU-hours) and allowing visualisation of usage information across the whole federation on a per community, per cloud, and per user levels.
- Availability and reliability monitoring of the clouds implemented by the EGI ARGO system. ARGO periodically tests the clouds with probes that are checking the proper functioning of the main functional pillars of OpenStack.

One or two EGI Cloud Compute service providers are used by each of the 5 Data Spaces ^3^. Multiple clouds can provide failover and scalability features but generates more complexity with respect to the synchronisation of scientific and user data, as well as the deployed applications. The cloud providers have been selected for each Data Space based on their functional capabilities (amount CPUs, GPUs, storage, network, etc.), and based on their matching interest in supporting the given field of science. This matching interest was typically ensured by having the country of the national cloud centre present in the research infrastructure that acts as data provider in the Data Space.

### 3.2 Data management technologies

The baseline storage at the cloud sites are file based systems. A subset, or the entirety of the scientific data have been copied from the primary storages of the scientific community institutes into these clouds. LOFAR, EMSO and SeaDataNet copied the entire datasets, equivalent of 9 TB, 20 TB and 38 GB respectively. GBIF copied a subset of its 13 TB dataset, and ENES copied a subset of its 50 TB dataset onto the EGI compute sites.

The Data Spaces used a mix of technologies for the data replication and access from their virtualised applications:

1. EGI Data Transfer service, based on FTS, was used to move a large number of files with verification of checksums and ensuring automatic retry in case of transfer failures. The solution fits for use cases when data from a single source has to be replicated to one or more additional locations.
2. The EGI DataHub service, based on OneData, was used as a high-performance solution that offers unified data access across globally distributed environments and multiple types of underlying storage. It fits for use cases when the primary copy of the data is spread across multiple locations, and a mesh of data transfers has to be implemented for the replication.

### 3.3 Authentication-authorisation technologies

The user authentication and authorisation have been implemented with the use of the EGI Check-in service. Check-in is a centralised solution that connects various Identity Providers with EGI service providers. The Identity Providers are federated in the EduGAIN network, complemented by providers of social login (Google, Facebook, etc.). The service providers are the compute centres and the high-level applications running inside them.

Check-in is one of the authentication-authorisation services that are compliant with the EOSC AAI interoperability standards. This enables the users of the Data Spaces to use the same identities both within EOSC and the EGI-ACE services.

## 4. Experiences

### 4.1. EMSO ERIC Data Space

EMSO ERIC (European Multidisciplinary Seafloor and Water Column Observatory, European Research Infrastructure Consortium) is a distributed research infrastructure consisting of ocean observation systems across the European seas. The main objectives of EMSO ERIC are to observe, perceive, and understand phenomena occurring on the seafloor and the water column over temporal scales that range from seconds to decades. To achieve this, EMSO ERIC deploys scientific instruments with physicochemical and biological sensors and develops specific tools to understand the obtained data. The EMSO ERIC consortium comprises 27 institutions from Europe and has 11 observatory sites with permanent instrumentation and 3 test sites where the research centres can deploy instrumentation prototypes.

Until 2022, EMSO ERIC's approach to data management involved a centralised system. Data were collected from different Research Facilities in various formats and were not harmonised. This data was then processed for harmonisation and re-ingested into a unified database. This process of data harmonisation and integration was facilitated by MOODA (Module for Ocean Observatory Data Analysis). MOODA, an open-source Python framework, is tailored for oceanographic data analysis. It allows for the opening and analysing of data files from scientific instruments, performing data quality control, and creating plots and data files in formats like netCDF and CSV. A key feature of MOODA is its focus on maintaining metadata alongside data, which is essential in oceanographic research for understanding the context and significance of the data.

Due to an increase in the variety of data types and sources from 2022 onwards, EMSO ERIC adapted its data management approach. Each Research Facility started to provide harmonised data directly, eliminating the need for central harmonisation. This shift was a response to the growing complexity and diversity of marine data being gathered across the consortium's expanding network of observatories. As a result, EMSO ERIC established its initial specification aimed at achieving harmonisation between EMSO data and metadata, with particular emphasis on key Essential Ocean Variables (EOVs). These specifications draw from previous initiatives such as OceanSITES, The NERC Vocabulary Server (NVS), and other standards, creating a catalogue of harmonised (meta)data, instruments, and other relevant information. Moreover, the metadata specifications are crafted to be interpretable by humans and machine-to-machine processes. This dual compatibility facilitates the development of tools that can automatically evaluate the harmonisation level of data objects and the adoption of FAIR (Findable, Accessible, Interoperable, Reusable) principles.

Furthermore, EMSO ERIC adopted ERDDAP (Environmental Research Division's Data Access Program) to manage and disseminate its data more effectively. ERDDAP is a data server that provides a consistent way to download subsets of scientific datasets in various file formats and make graphs and maps. This system is advantageous for handling diverse data types and sources, offering a more flexible and efficient means of data access and analysis. In particular, EMSO ERIC implemented a federated ERDDAP system. This innovation allows for access to data from all its ERDDAP servers through a single access point. This federated approach simplifies the process of accessing data from multiple observatory sites, ensuring that researchers and other stakeholders can easily obtain the data they need from EMSO ERIC's data space. By transitioning to a system where each Research Facility provides harmonised data and utilising a federated ERDDAP system, EMSO ERIC has significantly enhanced its data accessibility and usability. This advancement reflects the consortium's commitment to evolving with the needs of marine research and ensuring that its data infrastructure remains at the forefront of technological and scientific advancements in ocean observation.

Additionally, EMSO ERIC deployment of a machine-to-machine (RESTful) API to provide programmatic access to harmonised EMSO ERIC data and metadata. This interface allows authenticated users to access EMSO ERIC data and metadata through JSON-based requests. Other services include mechanisms for file-based discovery, access and integrity, and persistent identifiers. Improving search, access, and interoperability, such as more mature standardisation, better semantics, and standard metadata, enabled access to EOVs and multi-terabyte datasets of high-rate data (e.g. acoustic data), increased interoperability with other research infrastructures.

**Figure 2.**
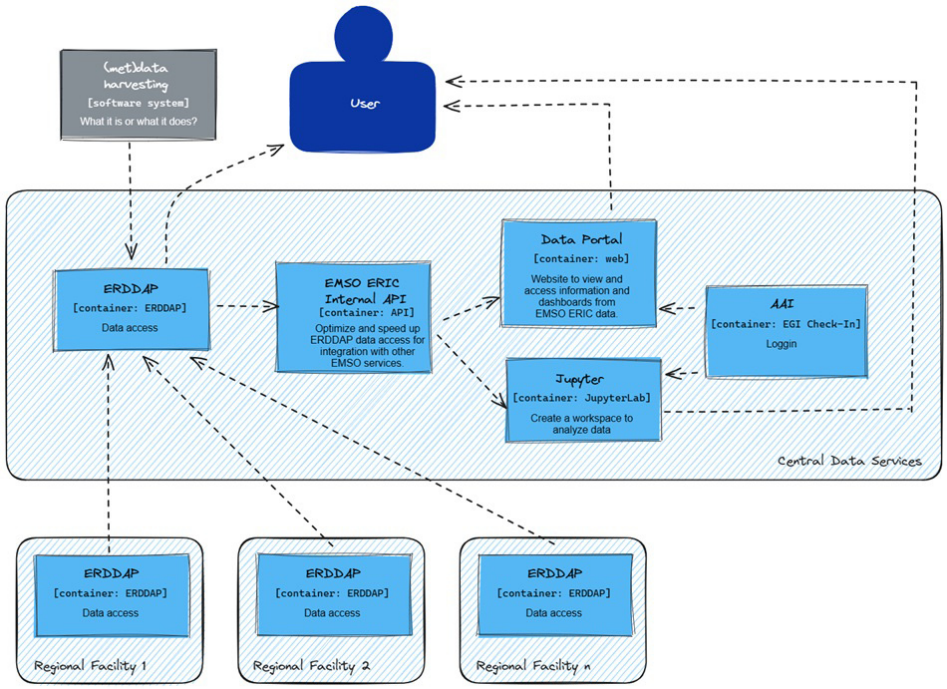
Overarching EMSO ERIC data management architecture.

The EGI-ACE project has allowed EMSO ERIC to enhance its data management capacity. This partnership contributed to the development of services and platforms that improved the data infrastructure at EMSO ERIC through the EGI compute cloud and services. As part of EGI-ACE, EMSO ERIC expanded its cloud infrastructure. This expansion (See Figure 2.) was aimed at increasing the redundancy and availability of data, which is vital for continuous scientific research. Specifically, the architecture was based on the following principles: (1) robustness and fault tolerance, including redundancy and failover capabilities on computing and storage resources, (2) scalability and security, including a distributed architecture for data access and analysis; and (3) preservation and archiving, including cold data storage ideally offering export capabilities. EMSO ERIC also incorporated Jupyter-based services provided by EGI resources. This addition improved the consortium's capabilities in data analysis and processing. Also, a federated identity system (AAI) based on EGI Check-in was implemented to simplify access to these services, improving the efficiency and security of data management.

The user interfaces and overall user experience have been enhanced through solutions developed with EGI-ACE. Open-access data and its integration with EOSC significantly impact researchers, educators, policymakers, and the general public across Europe and beyond. Access through the EOSC Portal and customised solutions have been received positively by users for their ease of access and practicality. The EMSO ERIC data portal received over 20K visits and more than 220K requests (from the API and ERDDAP federation) from over 3K distinct users in 122 countries. The countries with the most visits include China, Italy, Spain, the United States, France, the United Kingdom, Brazil, Germany, and Japan.

### 4.2. ENES Data Space

The higher resolution in Earth System Models is quickly resulting in enormous climate simulation outputs, creating significant challenges in data management cycles, especially to data sharing, processing, analysis, visualization, preservation, curation, and archiving ^4^.

Large-scale global experiments for climate model intercomparison have led to the development of the Earth System Grid Federation (ESGF) ^5^, a federated data infrastructure involving multiple modelling centers (data providers) around the globe and including the European contribution through the InfraStructure for the European Network for Earth System Modelling (IS-ENES) projects.

ESGF provides access to climate datasets from various efforts, such as CMIP (Coupled Model Intercomparison Project), which is now entering phase 7 (CMIP7) and is expected to produce more than 100PB of data of strong relevance for the IPCC (Intergovernmental Panel on Climate Change) reports.

Over the years, numerous efforts have been made to enhance computing and analytics capabilities near the ESGF data pools. The European Network for Earth System Modelling Data Space (EDS) ^6^ is a progression of these early initiatives, aiming to serve a broad user base. Specifically, the EDS aims to provide a comprehensive ecosystem that includes data along with a wide array of tools and services. This enables scientists to develop applications, analyse data, visualize results, track data provenance, reproduce analyses, share code, document workflows, and publish new products.

The development of the EDS has been driven by key needs identified through experiences with climate scientists. Traditionally, scientists would download climate datasets from central repositories (e.g., ESGF) to their desktop machines and process the data locally. However, the growing volumes of data have rendered this approach impractical. By providing scientists with large, ready-to-use datasets and integrated software solutions within the computing infrastructure, the setup time is nearly eliminated, significantly boosting productivity.

**Figure 3.**
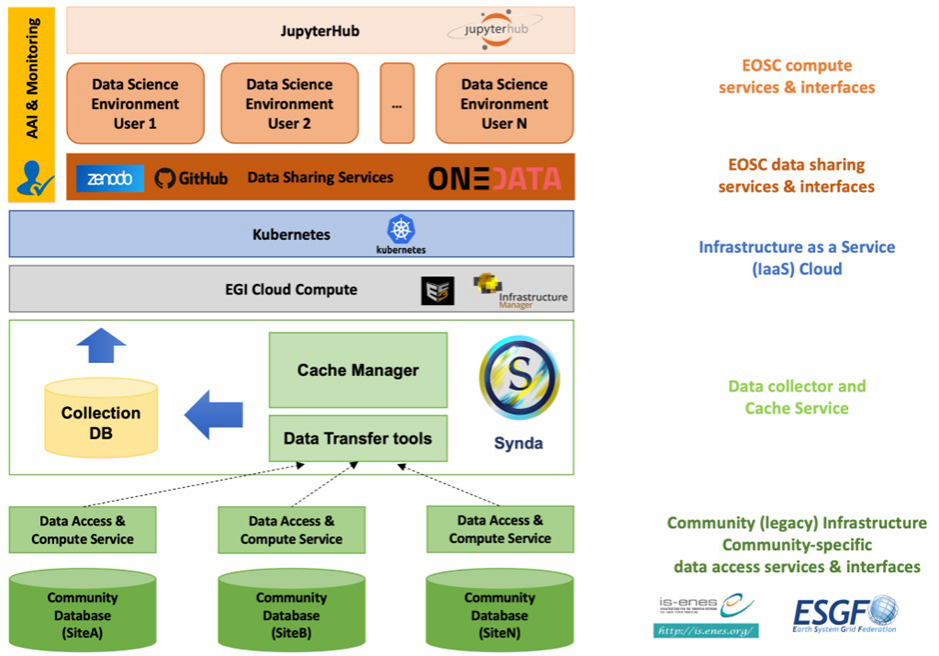
High-level architecture of the ENES data space.

Figure 3 presents a high-level overview of the EDS architecture, highlighting the main infrastructural components. The EDS web science gateway is implemented using JupyterHub ^7^. Authentication and authorization are managed through the EGI Check-in service, which is directly integrated into JupyterHub, offering users a seamless, secure login to services. The environment, based on the popular Conda package manager, includes a wide range of preconfigured scientific modules from the Python ecosystem. These support HPDA and AI-based applications in the climate domain and computing frameworks for parallel analysis (e.g., Dask ^8^, Ophidia ^9^). The TÜBITAK-ULAKBIM cloud site of the EGI federated cloud infrastructure provides resources for running the container-based services. The deployment leverages the Elastic Cloud Computing Cluster (EC3 ^10^) service, which manages and dynamically adjusts the size of a Kubernetes cluster on the cloud infrastructure. Additional details on the architecture design and implementation are available in previous work ^6^.

The EDS contains specific CMIP variable-centric collections sourced from the ESGF federated archive, meticulously curated and stored within its infrastructure. These collections encompass the most requested data across various models, experiments, and variables such as air temperature, precipitation, near-surface wind, specific and relative humidity. With diverse temporal resolutions, the data pool totals approximately 50TB. Moreover, users can request additional datasets to be integrated into the collection via a request form available on the data space portal.

Launched in late 2021, the ENES Data Space is accessible within the EOSC through the EOSC Marketplace. It offers a web portal with comprehensive information, tutorials, and training materials to help users navigate its primary features. Furthermore, it serves as one of the use cases in the Data Spaces radar under the Green Deal domain.

The ENES Data Space supported several use cases since its launch in 2022. These included research and academic activities in the climate domain, training events in key scientific conferences, support to Digital Twin applications, and scientific applications from the climate community. In total, over 70 users from 17 different countries joined the EDS. As a summary, users’ experience was rather good, the overall feedback was positive, and no critical issue was experienced from researchers working on the Data Space.

The EDS environment undergoes regular updates, incorporating new software releases, datasets, and tools. This process will continue as an organic expansion of the data space, particularly influenced by user feedback and requests. Current developments focus on implementing solutions for distributed processing across multiple virtual machines in a cloud environment, along with enhancing data access through the adoption of innovative technologies and associated standards and specifications.

After the end of the EGI-ACE project, the ENES Data Space is further supported and improved thanks to the participation in the EU-funded EOSC-Beyond project, where the data space will be one of the pilots, participating in the co-design and piloting activities of the new EOSC Core components. As a result, a new cloud provider of the EGI Federation, namely CESNET-MCC, has been already identified to provide the needed resource capacity. Moreover, in the context of the ENES landscape, the data space will be included as one of the compute services of the ENES RI AISBL, a no profit international association that is being established to foster the sustainability of the IS-ENES results and ensure the coordination of the ENES RI after the end of the project.

## 5. Conclusions and future work

We have created 5 international service setups for different disciplinary sciences from climate research through biodiversity research to astronomy. We call the setups Data Spaces because they gather data from multiple, independent institutes and serve this data to consumers, primarily to scientists in the academic sector through domain specific applications. Our setups integrate governance approaches, business models and technological elements from the EGI e-infrastructure federation, European research infrastructures and scientific communities. While the configuration and type of use cases our Data Spaces can support are limited compared to those foreseen by the sectoral Data Spaces in Europe, our pragmatic approach serves current demands in data intensive sciences, as indicated by the growing user base of our setups.

After the end of the EGI-ACE project the 5 Data Spaces are sustained by the providers based on a combination of in-kind contributions and EC grants. Because the main beneficiaries work in the academic sector, a move to a ‘pay-for-use’ model is not foreseen in the near future as it would impose additional, often hard to meet financial regulations on both the providers and users. The continuation of the virtual access funding to top up national capacities is therefore of paramount importance to a fully functioning cross-national service delivery system such as EOSC. Without the virtual access there is a high risk that service providers have to limit their operation to users of their own nation/discipline, significantly hindering the potential innovation impact of Europe. 

We monitor the European Data Spaces landscape as it matures, and we continue seeking for opportunities for collaboration and alignment with them on implementation approaches. The reuse of components and ‘building blocks’ could strengthen our impact, can stabilise operation and lower delivery and maintenance costs.

## Ethics and consent

Ethical approval and consent were not required.

## Data Availability

No data are associated with this article.
