# Supplementary material for: Scientific Data Spaces - Experiences from the EGI-ACE project
Source: Open Res Eur. 2024 Jul 9;4:136. [Version 1] doi: 10.12688/openreseurope.17418.1 (PMC11364964; doi:10.12688/openreseurope.17418.1)
Supplement: Peer review history

# Reviewer report: Reviewer response for version 1

**DOI:** 10.21956/openreseurope.18824.r42823

**Published:** 2024-09-06

**Authors:** Vicente Navarro

**Affiliations:** 1 European Space Agency, Villanueva de la Cañada, Spain

This open letter introduces EGI-ACE project view on the implementation of Data Spaces for research. Several domains have been addressed to demonstrate the transversal  character of the solution. In comparison with the DSSC ecosystem approach EGI-ACE architecture features a centralised coordination model.

 The open letter is well written and structured.

 The open letter taps into overall funding sustainability of the solution, however a move to a ‘pay-for-use’ model is not foreseen due to the academic nature of the users. This open point poses challenges and concerns on the continuity of the solution.

 Authors should consider looking into other similar initiatives run by ESA, like Copernicus Data Space Ecosystem https://dataspace.copernicus.eu/ , ESA Datalabs https://datalabs.esa.int/, GSSC Now https://gssc.esa.int/

Where applicable, are recommendations and next steps explained clearly for others to follow? (Please consider whether others in the research community would be able to implement guidelines or recommendations and/or constructively engage in the debate)

Yes

Does the article adequately reference differing views and opinions?

Yes

Are all factual statements correct, and are statements and arguments made adequately supported by citations?

Yes

Is the rationale for the Open Letter provided in sufficient detail? (Please consider whether existing challenges in the field are outlined clearly and whether the purpose of the letter is explained)

Yes

Is the Open Letter written in accessible language? (Please consider whether all subject-specific terms, concepts and abbreviations are explained)

Yes

Reviewer Expertise:

Big Data, AI, Data exploration and analysis, Data Science , Ground segment systems for science and spacecraft operations.

I confirm that I have read this submission and believe that I have an appropriate level of expertise to confirm that it is of an acceptable scientific standard.

# Reviewer report: Reviewer response for version 1

**DOI:** 10.21956/openreseurope.18824.r42824

**Published:** 2024-08-30

**Authors:** Maurizio Atzori

**Affiliations:** 1 University of Cagliari, Cagliari, Italy

The paper provides a concise overview of the EGI-ACE project, which has successfully set up and delivered five data spaces in various domains related to academic research. The article primarily focuses on two of these data spaces, maintaining a high-level description without delving into implementation details, making it accessible and easy to read.

 The paper clearly and convincingly justifies a centralized governance approach adopted by the project, contrasting it with the DSSC approach discussed in the related work (e.g., see Figure 1). This centralized model is applied to infrastructure and management while leaving the financial burden of operational maintenance to individual units, as opposed to a pay-per-use approach. The rationale behind these choices is rooted in the focus on data spaces within the academic research domain.

 However, the paper could be improved by further detailing the innovative aspects of the data spaces compared to traditional data repositories. While some advantages of the proposed infrastructure, such as access through federated EGI Check-in authentication, are evident, the paper does not sufficiently clarify how these data spaces go beyond being simple data repositories. For instance, a more in-depth discussion on the interoperability between different data spaces, their ability to facilitate cross-disciplinary research, and the potential for collaborative data management would highlight their distinctiveness. This would help underscore the innovative nature of these data spaces, differentiating them from conventional data-sharing platforms and providing a clearer understanding of their added value in the academic research landscape.

Where applicable, are recommendations and next steps explained clearly for others to follow? (Please consider whether others in the research community would be able to implement guidelines or recommendations and/or constructively engage in the debate)

Yes

Does the article adequately reference differing views and opinions?

Yes

Are all factual statements correct, and are statements and arguments made adequately supported by citations?

Yes

Is the rationale for the Open Letter provided in sufficient detail? (Please consider whether existing challenges in the field are outlined clearly and whether the purpose of the letter is explained)

Partly

Is the Open Letter written in accessible language? (Please consider whether all subject-specific terms, concepts and abbreviations are explained)

Yes

Reviewer Expertise:

NLP and Semantic Web (data interoperability); Member of the CINI Focus group on Dataspaces

I confirm that I have read this submission and believe that I have an appropriate level of expertise to confirm that it is of an acceptable scientific standard.

## References

[1] : Dataspaces: Concepts, Architectures and Initiatives. *https://ceur-ws.org/Vol-3606/paper49.pdf* .2023;

# Reviewer report: Reviewer response for version 1

**DOI:** 10.21956/openreseurope.18824.r42551

**Published:** 2024-08-23

**Authors:** Tuomo Tuikka

**Affiliations:** 1 VTT Technical Research Centre of Finland, Oulu,, Finland

Data spaces development is moving fast forward in industry, in organisations developing technological frameworks, and in many projects addressing the exploitation of data spaces in various domains. This specific paper has focus on scientific data spaces and presents approach by the EGI-ACE project for the setup and delivery of Data spaces for various scientific domains. Experiences from two data spaces are presented, EMSO ERIC, European Multidisciplinary Seafloor and Water Column Observatory, and ENES, European Network of Earth System Modelling data spaces.

 The EGI-ACE project started early in comparison to series of projects launched by the Commission in 2020 including domain specific Digital Europe projects and the Data Spaces Support Centre. As pointed out the project has introduced the first implementations of data spaces, and thus, through its examples and experiences, provides valuable and practical understanding on how to develop data spaces.

 Second chapter of the article reflects how the effort relates to Data Spaces Support Centre Blueprint (0.5 version) and presents also differences of the approaches. These two projects, however, are not easy to directly compare. EGI-ACE has a clear focus on open scientific data spaces and goal to implement them. DSSC, however, by its mandate is established to serve the interoperability of data spaces, both within and between them. Therefore, DSSC provides a generic overview of data spaces building blocks within Blueprint, but gathers solutions and implementations from its community of practice, these practically are the many domain oriented data space projects.

 About item (1) in related work, DSSC or the Blueprint 0.5 is not presuming equilibrium without planning, in fact, the activity of planning is necessary and the resources of DSSC are for use in planning action. To be fair to authors, design method was introduced later in Blueprint 1.0. Item (2) DSSC does not rule out sustainability via providers from EC, national and institutional funds, but perhaps the Blueprint 0.5 use cases tend to lean towards commercial approaches. Institutional funding is a valid approach, sustainability of the infrastructure is the key for success. Just a note on item (3), DSSC acts according to its mandate supporting those data spaces defined in the project contract. Furthermore, DSSC is not an implementation project, which is clear difference between EGI-ACE and DSSC. In this point authors compare projects with very different goals. (therefore partly)

 Architecture figure shows a very broad idea of how different stakeholders are mapped in both approaches. DSSC picture on the left expresses distribution of data by participants, with a connector software component as a mean to share data. While this picture is very simplified, it actually shows the different approaches which can be taken by data spaces. The difference is emphasized by the chapter 3.2 which explains how data is actually replicated from the primary storages. In general, by DSSC definition, ‘data space is specified as a distributed system defined by a governance framework, that enables trustworthy data transactions between participants while supporting trust and data sovereignty’. So the definition actually does not fix any specific form of governance, or technological choices. The data space developers can plan their steps to implement in their context. Eventually, in my opinion, interoperability is the key between the data spaces, which gives freedom to individual data space implementations to make their choices, as long as interoperability with others prevails if needed. This discussion goes beyond this paper focus though.

 Chapter 3 presents some practical innovations from the project, such as how the EGI approach has solved governance, and approach to funding, especially introducing virtual access units. Is there a way to depict technologies in chapter 3 (3.1, 3.2, 3.3) to help the reader?

 Chapter 4 explains the experiences with EMSO ERI data space which has an impressive consortium. The chapter focuses on data management approach and the shift to EMSO ERIC data management approach. As a reader I am assuming that in Figure 2 all the blue boxes are the result of expansion mentioned in the text, although I am not sure what has been the previous approach, which is expanded. Figure 2 has choices of font which make the texts almost unreadable in printed pdf format, on web page the figure needs to be zoomed. The latter is probably a feature of the web site.

 ENES Data space goes deeply into the implementation of the architecture, which is valuable to report. The amount of data is impressive. Furthermore, sustainability of the data space is addressed in the end, the funding model of the mentioned AISBL would be interesting to reader too.

Where applicable, are recommendations and next steps explained clearly for others to follow? (Please consider whether others in the research community would be able to implement guidelines or recommendations and/or constructively engage in the debate)

Yes

Does the article adequately reference differing views and opinions?

Yes

Are all factual statements correct, and are statements and arguments made adequately supported by citations?

Partly

Is the rationale for the Open Letter provided in sufficient detail? (Please consider whether existing challenges in the field are outlined clearly and whether the purpose of the letter is explained)

Yes

Is the Open Letter written in accessible language? (Please consider whether all subject-specific terms, concepts and abbreviations are explained)

Yes

Reviewer Expertise:

Data spaces, design and implementation

I confirm that I have read this submission and believe that I have an appropriate level of expertise to confirm that it is of an acceptable scientific standard.
